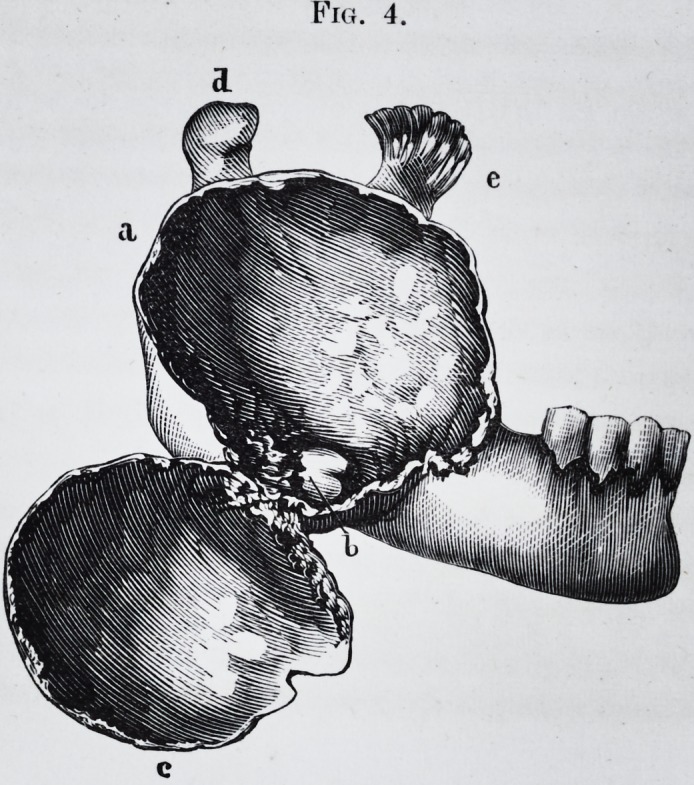# Dental Anomalies, and Their Influence in the Production of Diseases of the Maxillary Bones

**Published:** 1860-04

**Authors:** A. M. Forget


Plate 1.—Fig. 1.

Fig. 2.

THE

AMERICAN JOURNAL

OF

DENTAL SCIENCE.

Vol. XI. NEW SERIES.—APRIL, 1860. No. 2.

### ORIGINAL COMMUNICATIONS.

### ARTICLE I.

*Dental Anomalies, and their Influence in the Production of Diseases of the Maxillary Bones.* By A. M. Forget, M. D. (Translated for the American Journal of Dental Science.)

[Continued from the January Number.]

*Anatomical Examination of the Tumor.*—These remarks would be in incomplete, if to the surgical result which has up to the present moment especially occupied my attention, I did not add the anatomical examination; considered then in reference to the soft parts which surrounds it, it is ovoidal in form, being thirty centimetres around its greatest circumference, and twenty-three around its least. The soft parts adhering to its external face are furrowed by several fistulous canals terminating at the inflamed and ulcerated points of osseous tissue. This is thin, soft and depressible, and is pierced by holes which conduct to the interior of the cyst, and from whence a purulent, thick, viscous liquid, of a reddish color, percolates. A probe introduced by one of these openings is soon arrested by a hard, persistent body, which gives a sound in percussion similar to that of compact tissue denuded of its periosteum. In order to show this body uncovered, I raised the tissue of the gums which formed a kind of operculum at the superior part, thus completing the cyst in which the morbid product was shut up.

This dissection made, the anatomy of the tumor, with a complete revelation of its nature, was revealed. All of that portion of the left side of the inferior maxillary comprised between the ramus of the jaw and the first small molar was found to be converted into a cavity which contained a hard, oval mass, of the size of an egg, having an uneven surface, covered by small tubercles which were invested by a layer of enamel penetrating into the substance of the bone, and easily recognizable by its shining appearance and its peculiar color, (pl. 1, fig. 1.)

Having divided the tumor into two equal parts, it was ascertained that it consisted of homogeneous compact tissue, of the consistence of ivory, and of a grayish-white color. A kind of regular arrangement of the elements which composed it could also be seen by the naked eye, (pl. 1, fig. 1, and pl. 2, fig. 1.) Between the tumor and the osseous parietes of the cyst was a thick membrane, apparently of a fibro-cellular structure ; this was free on the side next the tumor, of which it covered the intramaxillary portion, (the part covered by the gums having been removed,) while it was connected to the cyst by filamentous prolongations of a cellulo-vascular character, reaching to the numerous openings on its exterior. The external surface was bathed by a muco-purulent fluid, having a fetid odor similar to that of a carious tooth. At the anterior extremity of the base of the tumor a depression was remarked to which the crown of a molar tooth was attached, wedged in between it and the maxillary bone. This

Plate 2.—Fig. 1.

Fig. 2.

arrangement is represented in pl. 2, fig. 1, c, where a portion of the bone has been cut away; in the same plate is shown the second small molar lying obliquely in the substance of the jaw bone, (pl. 1, fig. 2, d,) under the alveolus of the first small molar, e.

Thus we have discovered the situation of all of the teeth with the exception of the two last molars; the alveolar spaces which are intended for them being occupied by the tumor. The question now naturally arises—what has become of them? The explanation that their bulbs were originally compressed by the pathological production, that these atrophied and finally disappeared, leaving no vestige of their existence, is not permissible, in view of the numerous cases which demonstrate the simultaneous development of the teeth and abnormal products in the same maxillary bone. In the analogous cases which I have had occasion to examine, all of the teeth have possessed their ordinary dimensions; although frequently deviating from their normal direction, often far removed from the place they should physiologically occupy, and sometimes enclosed in the substance of the morbid growth, they have yet, nevertheless, always been found. Thus their absence, as due to atrophy and to the re-absorption of their primordial elements, does not seem to me to be founded on a sufficiently demonstrative fact.

As to the present case, it does not appear necessary to invoke the aid of a merely seeming interpretation; for the tumor, studied in its origin, its mode of development, its symptoms, its seat, and above all, in its anatomical characteristics, refutes the idea. The microscope, in short, in this instance, is in agreement with the direct intuition that it is composed of the same substances that form an integral part of the teeth, (pl. 2, fig. 2.) Now, to my mind, this is sufficient to reveal the origin and nature of the tumor; its origin, however, is proved by M. Ch. Robin's investigations which have been textually reproduced in the description of the plates. I do not hesitate to say that while the nutritive juices were forming the two molars in question, according to physiological order, each separately, hypersecretion from morbid influences intervened and the aggregation of immature dental elements took place, thus producing the monstrosity.

If we now proceed from the study of the material characteristics of the disease to the first influences which prepared the way for its invasion and, favored its development, we shall find in the presence of the fibrous membrane which formed a covering for the tumor, an indication which may enable us to fix a point of departure, and also to comprehend the mechanism of its formation.

The membrane in fact, limited to the portion of the tumor contained in the interior of the cyst, or intramaxillary portion, establishes between this and the portion jutting above the osseous or extra-alveolar edges, a decidedly marked line of demarcation. It is simply, according to my views, the exaggerated reproduction of a normal anatomical tendency existing under a form and in very restricted proportions when the evolution of the teeth proceeds regularly. I speak of that membrane which envelops the dental root, designated by most writers under the name of alveolo-dental periosteum, and described with much precision by my learned confrère, M. Oudet, under that of the cortical membrane, because to it is attributed the function of secreting the cortical substance or cement. This membrane is in reality the external layer of the dental follicle, under conditions of aspect, form, structure and vitality produced by disease. Hence, from the morbid activity of the secreting organ, the abnormal formation of ivory and that of the osseous matter or cement, and the regular composition of the dental ostoid, I am enabled to assign an origin to this tumor without exceeding the limits of a severe induction.

I think that there had been :—

1st. An original union of the follicles of the two last molar teeth; whatever cause produced it, so intimate a fusion existed between them that they were one.

2d. That under the same morbid influence, the excess of vitality of the different organic elements of these follicles produced the hypersecretion of the ivory and osseous substances.

3d. That the diffusion and irregular aggregation of these constituted the pathological formation.

4th. And finally, this last, by its development and the volume it acquired, has had the effect of producing the excavation or cyst in the centre of the jaw; at the same time it kept up an inflammation, which, after having disorganized the osseous tissue and greatly altered the structure of the surrounding soft parts, it left no other resource than the operation I subsequently performed. The consecutive result of the operation only confirmed that obtained in the first instance. Two years after the operation the right half of the maxillary bone was found to be depressed backwards and towards the median line. The point of the chin was effaced by the inclination to the right; the inferior dental arch was behind that of the upper jaw, nearly a centimetre: this gradually diminished and became hardly perceptible at the level of the two large molars. This defect in the parallelism between the two jaws almost disappears when the elevator muscles contract with a certain degree of energy, so that mastication is not impaired; it increases, on the other hand, when the jaw is lowered. Perhaps it may also be added, that the mouth being open, the deviation of this bone from right to left determines a similar deviation of the floor of the mouth, of the tongue and of the vault of the palate and its pillars, without impeding the regular performance of their functions. And as to the portion of the left branch of the jaw which remained in the wound, I am assured, that forced by the contractile action of the temporal and external pterygoid muscles, it was carried above and inside the zygomatic arch, where it remained immovably fixed. I proved, moreover, the existence of a band of cicátricial tissue, extending along the track of the incision of the soft parts and uniting the two osseous extremities. This extent of inodular tissue, being endowed with great firmness, offered an important *point d'appui* to the tongue in its different motions. This tissue, in becoming more perfectly organized, re-established the continuity of the bone in a certain rudimentary manner and enabled it to act, with more and more precision and force.

If, in the preceding remarks, I have said that the case is without analogy in science, I have not intended to ignore the existence of various anomalies of nutrition and dental development which have been described, nor the histological relations that exist between these and the anatomical condition exposed. Numerous and varied as these anomalies are, for the most part, being constituted by the lateral and most frequently by the partial union of two or more contiguous teeth they do not affect them in form, position or functional aptitude: nor does the maxillary bone which holds them receive any morbid taint. Now the difference between these slight anomalies and those which are distinguished by the complexity and development of the elements which compose them, is easily seen, and at least in a surgical point of view, all serious analogy must be abandoned; I say, in a surgical point of view, because anatomically, the unity of composition and the law of formation of primordial tissues, indicate from an elementary to a complicated fact, a common teratological influence and a necessary affiliation based upon a series of intermediate facts which may be considered as steps by which the organism successively passes from the simple to the complex. From this unitarian doctrine, so learnedly generalized and applied by its illustrious author, Geoffroy Saint Hilaire, to the philosophical study of the dental systems of mammifers and birds, the partial fusion of two or more teeth, either by their crowns or by their roots, may be looked upon as a fit point of departure for the anomaly which I have represented in entirely isolated proportions; they are, so to speak, the first step taken by nature outside of regular physiological development, and a tendency towards the bizarre and truly monstrous case I have brought forward. However, considered either in its initial type or in its more advanced form, this anomaly is not elucidated without duly estimating a primitive morbid state of the odontogenous follicles; since, when deprived of the vascular element, as remarked by Oudet and others, the dental substances are incapable of being the seat of nutritive or pathological operation. Hence it is evident that to give an account of the alterations of structure and form here presented, we must study these in their origin, for thus only do we perceive their connection to the organism under whose laws they exist. Now, whether we consider that anomaly as the result of disease of the follicles, or with Geoffroy Saint Hilaire, as a simple departure from the usual work of physiological growth, the grouping of the dental elements into an amorphous mass in the human species, it will be better understood, if according to the learned author of the theory of analogies, we repeat what takes place at the origin of the evolution of the teeth:—"Each dental rudiment," says the illustrious naturalist, "is surrounded by an exhalent envelop which stimulates the growth of the tooth. And while the latter are isolated, they grow and form teeth in a manner very easily understood; but if two osseous nuclei approach and touch each other during this exhalation of nutritive juices, they unite, crystallizing like true stalactites, and form a compound of several dental elements. In this manner the molar teeth are constituted; and likewise those agglomerations of teeth, which in the elephant and many other ruminants, represent the normal state: this is exceptionally observed in man."

Saint Hilaire had one of those cases in view when he formed the opinion I have quoted; he has given a drawing of this anomaly in his appendix to the dental system of mammifers and birds. It will be observed that there are certain points of analogy between this one and the one mentioned in the preceding remarks.

*Case II.—Tumor Occupying the Alveoli of the two Bicuspids.*

The subject of this observation was brought before the Society of the Faculty of Medicine, in 1809, by M. Oudet. He complained of the great impediment caused by a tumor in the mouth ; it was found to consist of a large mass which had been mistaken for tartar, extending above the dental arch and producing a decided eminence on the side of the cheek. Extraction was performed and then the nature and form of the tumor became appreciable. From M. Oudet's description it appears that it resembled a cone implanted by its apex in the alveolar cavity, its base being in contact with the crowns of the contiguous teeth ; it was formed by an aggregation of the dental elements appertaining to the bicuspids, which by their arrangements reproduced the form of the milk incisors and canines.

In this case the pathological influence had also effected an exaggeration of the elementary parts of the bicuspids, and in irregularly associating them between them it had brought them to the condition of a single tooth. Other intramaxillary tumors exist, which are no more due to the original and morbid aggregation of the different dental elements than those with which I have been occupied, but to a secondary hypertrophy of one or more of these elements. The tooth presenting this anomaly of nutrition generally preserves its chief physiological characteristics, and the alveolus, which is its receptacle, is not sensibly modified in its form, as long as the tumors, which are nearly always attached to the dental roots, are confined to a very small compass ; thus, for the most part, they are inoffensive. It is when they grow and become large enough to change the conditions of vitality and the appearance of the jaws that these tumors necessitate a resort to urgery.

Such a case was observed by my colleague, M. Maisonneuve, under the following circumstances, which he was kind enough to communicate.

*Case III.—Osseous Intramaxillary Tumor attached to a neighboring tooth. Simultaneous evulsion of this tooth and the Tumor.*

A man aged forty-five years, came from a province to Paris, in order to have a tumor removed from the inside of his mouth, where it was the cause of much distress. This tumor occupied the left side of the lower jaw, and formed a decided protuberance from this in both sides, but particularly so on the outside where it resulted in giving a most unpleasant expression to the face. At the small extremity of the ovoid, represented by the tumor, the incompletely destroyed crown of a carious tooth was visible, but this was partially marked by the elevation of the gum, caused by the morbid growth contained in its alveolus.

Before operating, M. Maisonneuve directed his patient to have the carious tooth removed, thinking thus to open a way by which he might the more easily explore and take away the encysted product. This preliminary operation, however, had an unexpected and decided result, for the tooth, and with it the tumor which was annexed, were removed by the same effort. This last, which was about the size of a large pigeon's egg, was fastened to the tooth by a very narrow pedicle; this being opened along its axis, the line of intersection between it and the root could be determined. Microscopical examination demonstrated that the specimen contained no ivory, but was composed exclusively of osseous tissue.

As to the patient, he was not slow in recovering; it followed, after this double extraction, that the parietes of the alveolus, separated and raised by the tumor, that a slight inflammation visited the internal surface of the cyst which contracting upon itself, finally resulted in its complete shutting up.

Comparative anatomy has proved that morbid productions, analogous to the one which has just preceded, are met with in animals, and especially with the large ruminants. M. Goubaux, Professor in the veterinary school at Alfort, has published examples of some, and I give one below,* which has the double interest of showing at the same time the anomaly of nutrition which causes it and the alteration of the maxillary bone, which was its effect.

*Case IV.—Encysted Osseous Tumor in the Upper Jaw of a Horse.*

This tumor was found on the interior of the alveolus of the upper canine tooth, to which it had contracted no adherance; it was encysted and very irregular in form, and about as large as a hen's egg. A microscopic examination, as in the preceding case, showed it to be composed entirely of osseous or cemental tissue.

I should not omit to say that these morbid growths are all invested by a membranous envelop which is found to be the alveolo-dental periosteum. This membrane secretes the osseous elements constituting, by their aggregation, these intraalveolar tumors, wrongly viewed as dental exostosis; to look at them, in fact, in this point of view, is to give them physiological explanations which properly belong to the osseous system. It is to state that they owe their origin to the tooth itself, whilst in reality they are a substance entirely distinct in itself, and thus owe their formation and growth to organic operations to which the tooth is entirely foreign.

This absence of all original connection between the tooth and these osseous products of accidental formation, is com-

*** I am indebted to the kindness of M. Leblanc, member of the Academy of Medicine, for this specimen.**

pletely proved by the last case, which shows this product as isolated and without any relation of continuity of tissue with the canine of the horse to that with which it was simply brought in juxta-position.

There is another fact for which I am equally indebted to comparative pathology, and which enables us to appreciate the very considerable degree to which the simultaneous hypertrophy of dental substances is carried, and the gravity of the disorders it creates in the bone where it is observed. This case was communicated by M. Boulay, of the school at Alfort.

*Case V.—Rare case of Dental Hypertrophy in a Horse.*

The disease occurred in the second grinder of a horse which died of glanders. The animal had a large growth on the right side of the upper jaw, in the middle of which this tooth had, by its unusual development, produced an extended inflammation, characterized by the rarefaction of the osseous tissue, and the enlargement of its spongioles. The tumor was very irregular in form and weighed about one kilogramme. It was caused by simultaneous hypertrophy of the dentine and cement, but chiefly by the hyper-secretion of the last. This substance is deposited in successive layers of unequal thickness on the surface of the tooth, where it forms crimplings or swellings, separated from each other by circular grooves; this arrangement explains how some teeth are fastened to the interior of the jaws, and gives a reason for the impossibility of extracting them without breaking the dental arch which embraces their depressions, or is nicely adapted to their abnormal swellings.

A very interesting peculiarity of this case is the existence of cavities within the tumor, containing fragments of osseous tissue, which are movable. These are manifestly parts of the spongy tissue of the jaw. Microscopic examination leaves no doubt as to the osteo-dentine character of the formation.

We think it unnecessary to prove, that in man, as in animals, it is very rare for the maxillary bone not to become affected by the presence of altered or deformed teeth. This has been observed by M. Boulay, in his remarkable memoir on the diseases of the teeth of herbivorous animals.

I cannot terminate this part of my work, particularly devoted to the study of osseous lesions caused by anomalies of nutrition in the teeth, without adding an example of partial necrosis of one of the intermaxillary bones, produced by the premature growth of two second incisor teeth, in a child three years of age. I am indebted for it to Dr. Géry, Sr.

*Case VI.—Anomaly of Nutrition in two Second Incisor Teeth in a Child three years of age. Necrosis and Loss of a considerable portion of one of the Intermaxillary Bones.*

This child, having a tumefaction of several weeks growth in the gums of the incisor teeth of the upper jaw, which was painful to the touch, was placed under the care of Dr. Géry, who prescribed lotions with a solution of alum. In a few days the inflammation and swelling subsided. It was then easy to examine the teeth, which were movable, and to discover that a purulent fluid exuded from their alveoli. The teeth fell out spontaneously in a few days, and the existence of voluminous and loose sequestered bone was ascertained. This was easily extracted.

I examined the specimen with the greatest care, and I am satisfied that it consisted of nearly the whole of one of the intermaxillary bones. It presented an unequal, rugose surface, and is creased by numerous vascular canals. The alveoli of the two temporary incisor teeth, and above them, two cavities in the substance of the bone surrounding the permanent incisors, of natural size, may be easily seen. I think, with M. Géry, that the premature evolution of the germs of the second dentition was

Plate 7.—Fig. 1.

Fig. 2.

the cause of the affection. The absence of all proportion between the dimensions of the alveolar arch and the age of the patient and the volume of these teeth abnormally developed, furnish the reason for the ulcerative inflammation which caused the dropping of the milk teeth and the deep seated lesions which compromised the bone itself.*

### CHAPTER SECOND.

### *Anomalies in the Position of the Teeth: their Pathological Consequences.*

In order to achieve the study of the histological relations existing between dental anomalies and the diseases of the jaws, it remains for me to consider the anomalies in the position of the teeth, which, like those of nutrition, already discussed, are the point of departure of a certain number of pathological phenomena.

These anomalies may be divided into two classes. In the first, we have the tooth deviating from its natural position within the limits of the dental arch; in the second, the tooth is found at some point of the jaw, more or less removed from the dental arch. I have given some examples of these in pl. 7, figs. 1 and 2; and pl. 8, figs. 1 and 2. Thus in pl. 7, fig. 1, the teeth occupy their alveoli; while in pl. 8, fig. 2, they are at some distance from them; the supplementary canine tooth, particularly, may be observed encased horizontally in the floor of the nasal fossæ.

We find in the *Bulletins de la Société Anatomique*† an example of a superior incisor tooth received into an accidental cavity, made between the two upper maxillary

* This case, it seems to me, settles the question now before the Academy of Sciences, as to the existence of the intramaxillary bone: according to M. Rosseau, who has found a sincere opponent in Dr. Larcher, this bone does not exist. The above observation proves the correctness of Dr. L's position.

† Tome 11, p. 25, obs. by M. Lacroix.

bones. This cavity resembled a median maxillary sinus, seven or eight lines in diameter. The report does not state whether this morbid condition was revealed by exterior signs; but it is easy to comprehend that so serious a development should eventually require the surgeon. It is true that these anomalies are sometimes unsuspected during the life of the subject affected, manifesting such weak objective signs as hardly to be considered as pathological phenomena. Most of them, however, are a cause of incessant irritation, causing caries of the regular teeth, caries of the alveolar border, and fistulous abscesses. See pl. 8, figs. 1 and 2.* In some instances the only indication of the existence of these anomalies is to be found in the persistence of dental neuralgia, so acute as to result fatally. See the case reported by Dr. A. Desirabode in the *Journal des Connaisances Médico-Chirurgicales.*†

*Case VII.—Anomaly of Position of a Wisdom Tooth. Acute Neuralgia. Death by Suicide.*

The subject, named Chéron, and a wheelwright by profession, was born at Villefranche, in the year 1816. In 1841 he was taken to the hospital *de la* Pitié, then under Lisfranc. Having been subject for some time to violent toothaches, he precipitated himself from his window. On the day after his entrance, he died from the effects of the fall. At the autopsy the lower left wisdom tooth was found under the much tumefied gum, situated with its crown pressing against the last molar, and its roots corresponding to the base of the coronoid apophysis.

In regard to this case, there appears to be no doubt that the suicide of the subject arose from the exasperation pro-

* The memoir of Dr. Toirac, in the ***Revue Medicale*** for 1824, may be profitably consulted in the study of this subject. It is entitled, ***Les deviations de la dernière molaire et les accidents qui peuvent accompagner sa sortie.***

† Number for the 1st of September, 1851.

Plate 8.—Fig. 1.

Fig. 2.

duced by the dental pains. The tetanus, also which intervened before death, came from the same cause. I may add further, that the other three wisdom teeth were regularly developed.

I remark above that the teeth sometimes migrate from the alveolar border. I cite, in support of the statement, a case of Prof. Blandin,* wherein it appears that a deviation from the regular position may cause more or less large tumors of the jaws.

*Case VIII.—Anomaly of Position of two Molar Teeth, Causing a Tumor in the Vault of the Palate. Cancer of the Maxillary Bone suspected. Error of Diagnosis discovered during the Operation.*

A woman, aged forty-three years, entered the hospital Beaujon for a disease dating back eighteen months. It was characterised by fungous ulcerations occupying the nose and right cheek. It presented a tumor of the form and size of a nut, upon the left side of the palatine vault, limited without by the dental arch, and within by the median line, and extending antero-posteriorly from the neighborhood of the canine tooth to the veil of the palate. Struck by carcinomatous aspect of the ulcerations, Blandin formed an unfavorable diagnosis. He decided, in treatment, upon the ablation of the tumor, and the cauterization of the facial ulcer. Having determined, a crucial incision was made upon the tumor, the flaps were dissected back, and the surgeon was already disposed to attack with chisel and mallet, when, having sponged the wound, he perceived *a shining, white body* in the centre of the tumor; upon touching it he perceived that it was movable, and it was extracted. It was a molar tooth with three short roots, but having the form and size of a first large molar.

* *Des Dents.*

A second tooth was extracted; it was as large as the first, and also multicuspid. "Great was my surprise," says Blandin. These teeth had pierced the internal part of the alveolar border, and lodged between the mucous membrane and the corresponding osseous plate. The wound of the palate was cauterized to staunch the flow of blood, the ulcerations of the face were also several times cauterized, and two months after, the patient, cured, left the hospital.

Were these ulcerations of the face cancerous? Blandin re-affirms it in concluding his report; to him it was a double disease. This view is open to controversy. Indeed so rapid a cure of an ulcerous cancer would appear very rare and improbable.

Be this as it may, the important point of our theory is established by this case; viz. that tumors caused by the aggregation of several teeth removed some distance from their normal place of development, may exist in the substance of the maxillary bone. A tumor, somewhat like the preceding, occupying a part of the lower jaw, remote from the alveolar border, would have been taken away, according to Blandin, by Marjolin and Duval, if he (Blandin) had not pointed out the error in their diagnosis in time. These facts suffice to show how important it is for surgeons not to ignore these migrations or changes in the position of the teeth, in forming diagnoses of tumors in the vicinity of the dental arch.

If, as in Case viii, the turning of the tooth from its regular position by this variety of anomaly only exceptionally constitutes a serious pathological phenomenon, then, when it is removed some distance from the other teeth, it does not oppose their regular evolution; in being removed the case is different from that in which the tooth still preserves its place in the arch, though developed in the vicious attitude entailing changes in its natural relations with the contiguous teeth and the maxillary bone. One may easily perceive that the tooth thus deviating becomes a cause of active and permanent irritation, which may eventuate in lesion more or less grave in character.

*Case IX.—Anomaly in the Position and in the Development of a Wisdom Tooth. Penetration of one of the Roots into the Dental Canal. Medullary Osteite of the Ramus of the Jaw. Resection and Disarticulation of one of the Condyles.*

The patient, a man, aged twenty-six years, had been affected for some time by very acute dental neuralgia, corresponding with the last molar teeth of the right side of the lower jaw. The tumefaction over the ramus of the jaw gradually increased over the region of the masseter, and finally rendered lowering of the jaw impossible. Hard, irreducible under pressure, the swelling was evidently the result of hyperostosis.

The patient now decided to enter the hospital *de la Pitié*, under the care of M. Maisonneuve, who, after having laid bare the tumor, applied the trepan, in the hope of finding the tooth which he presumed to be the cause of the disease. This operation proving unsuccessful, he resected the bone near the first large molar, and disarticulated the condyle.

The specimen having been sent to me by my honorable colleague, I was able to establish all the characteristics of an osteite from the various degrees of development. Cut along its axis, the branch of the bone presented several cavities in its substance, carpeted by a pyogenic membrane bathed in pus. Some of these cavities were completely closed by osseous tissue, and some opened exteriorly by passages made by the ulceration ot the tissue itself. One of these cavities, occupying the condyle of the jaw, opened through the articular cartilage. The development of these encysted abscesses could not have been carried on without producing rarefaction of the areolar tissue of the bone, and the simultaneous swelling of the compact layers; this double action determined the augmentation of the ramus, and the accruing pathological vascularity which was revealed by numerous osseous canaliculi with which its tissue was perforated.

The medullary osteite, of which this specimen is a striking example, terminating by suppuration and elimination of sequestres and splinters from the purulent cavities I have described, effected a less rapid progress at the angle of the jaw where the hypertrophic condensation of the osseous elements exist in considerable proportions. The trepan was here unsuccessfully applied. As to the cause of the disease, I do not hesitate to attribute it to the abnormal growth of the wisdom tooth enclosed in the base of coronoid apophysis, and passing nearly a millimetre over the borders of the alveolus in which it was situated. This, like the dental crown which fills it, has twice the dimensions it should have under ordinary circumstances, and the tooth in leaning against the neck of the adjoining tooth, (the second molar,) displaces it in order to ascend. Hence it is obliged to effect its development in the substance of the bone.

A section of the jaw under the dental canal, demonstrated the existence of a communication between the canal and the alveolus of this wisdom tooth. The roots were far from healthy, their points being truncated and unusually dilated where they projected into the canal; the bluish discoloration also plainly indicated an alteration in structure. Other cases, furthermore, favor my view of the etiology of the preceding.

The first of these, an illustration of which will be found in pl. 6, fig. 1, has been published in my inaugural thesis, but I shall here only give those details that relate particularly to the subject of this work.

CASE X.—*Osseous Cyst in the substance of the Jaw.—Anomaly in the Development of the Wisdom Tooth.*

Madam D., aged thirty, of strong constitution and enjoying good general health, entered the hospital *de la Pitié*, in the month of April, 1838. She was affected with a swelling on the right side of the jaw, of the size of a large hen egg. It was bounded in front by the second incisor, and behind by the base of the coronoid apophysis. As to the origin and progress of the disease, the patient stated, ten years before, after cleaning her teeth which had always been diseased, she became conscious of a small tumor developing itself near the molars; it gradually increased and occasioned very severe pains, which she attributed to the teeth, some of which fell out.

The extent of the tumor decided Lisfranc to resect that portion of the jaw. The operation was speedily followed by cure, and Madam D. left the hospital six weeks after.

Upon examination the piece removed showed considerable induration of the soft parts about the maxillaries, and under these, of the development of the jaw, in the centre of which was a large cavity filled with a sanious and purulent liquid. This cavity was formed by the two plates of the maxillary bone which were very thin, being in some places reduced to the thickness of the periosteum. Its floor, constituted by the base of the enlarged bone, presented the crown of the wisdom tooth in relief in the cyst. It was pushed horizontally from the base of the coronoid apophysis, solidly enclosed in the calcareous tissue: pl. 6, fig. 1. Its abnormal position and the regular development which it effected in this vicious situation, left no doubt as to the part it had played in the successive production and evolution of the disease of the bone. It followed in short, from the examination, that the wisdom tooth could not grow without exercising the continual pressure against the neighboring tooth, giving rise to the pains of which the patient had spoken: this pressure also occasioned the inflammation of the gums, the caries, the loosening and spontaneous falling out of most of the other teeth.

Two other cases, not less interesting than the last, have been reported to me by my *confrères* MM. Nélaton and Maisonneuve. Though differing as to the nature of the lesion which necessitated the intervention of art, they resemble each other in the original morbid proclivity which had been the point of departure.

*Case XI.—Cyst in the Ramus of the Lower Jaw, coincident with the presence of a Molar Tooth in its Cavity.*

The patient was a woman aged about thirty years. Her cheek had slowly but progressively tumified from the effects of an abnormal development of the ramus of the jaw. The tumor was accompanied by dull pains. It finally took the form of a nearly regular half sphere. The ampliation of the lower jaw was chiefly effected by the hemispherical raising of the external table, the internal preserving its regular position. M. Nélaton decided with reason to attack the tumor with the bistoury and probe; in this manner the exterior soft parts having been conveniently incised, the external wall of the cyst was circularly resected. This once removed it was easy to explore the cavity, and the result was the discovery of a tooth situated in its lowest part, pl. 4, fig. 4, *b*. The tooth, partially enclosed in the calcareous tissue, was taken out after several trials. The operation was an entire success; for twelve years after the disease had not returned.

The preceding cases it seems to me, are calculated to throw some light on the origin of certain cysts which are frequently found in the substance of the jaw, and which it has been the tendency of recent researches to attribute to the abnormal increase in the natural cavities of the organism; that is to say, to the growth of the dental follicles themselves. This opinion is founded as well upon the consideration of the seat of these tumors, which appear along the dental arch, and at an age corresponding generally to one of the two phases of life characterized by the double work of dentition, as upon the microscopic results which has proved the existence, several times, of an epithelium investing the interior of these cysts, on the roots of the teeth prominent in them.* Now as to the epithe-

* Case by Dr. Dēnucē. *Bulletin de la Soc. Anat.*, vol. xxii, p. 806.

lium, does it not indicate that a physiological tissue, serving to invest a normal cavity, must also contribute to the development of the encystic tumors, and in some sort serve as a bases to their definitive constitution. As a partizan, in pathogenesis, of the doctrine of naturalism, which sees but an infraction of a primitive organic type in most morbid growths, I accept this interpretation as it is demonstrated to me by the existence of alveolar cysts in direct communication with the dental follicle.

The last case I shall cite, supporting this view, is complex; it exhibits two distinct pathological conditions; the primitive or original is that of a dental cyst with an epithelial investment; the other, or consecutive, is that of the inflammation of the lower jaw rendering disarticulation necessary.

*Case XII.—Dental Cyst. Inflammation of the Lower Jaw Bone.*

A young man aged twenty-seven years, consulted several surgeons of Paris in reference to a swelling of the right side of the lower jaw which had acquired three times its proper dimensions. Nearly all the time tormented by a dental neuralgia, which had announced itself twelve years before, the patient at its first symptoms, had remarked a tumefaction about his molar teeth. The tumor did not make any appreciable progress until after a period of three years, when its evolution was marked by continued, but moderate pains. The body of the jaw on the right side was so deformed as to represent an irregular ovoid with some points in relief; the gums were red, thick and tumefied, bleeding with extreme facility, and the teeth, deviating for the most part from their proper direction, were more or less movable. The submaxillary region presented some engorged lymphatic ganglions. M. Maisonneuve, having charge of the patient, deemed disarticulation necessary; this he performed about the middle of April, 1857, but it was followed eight days after, by the death of the patient, from gangrenous inflammation of the wound and secondary hemorrhage.

The disease in this case, was, during the life of the patient, taken successively for a cyst, a cancer, and finally for an inflammation of the bone.

Subsequent examination determined that the case was one of complex lesion. It revealed a large molar tooth, situated horizontally in a cavity in the centre of the tumor, which had thus viciously placed, regularly developed, pl. 5, fig. 1, and pl. 6, fig. 1. This tooth was surrounded by a fungous growth, which, under the microscope, seemed to be formed of fibrous or fibro-plastic tissue, with an epithelial investment; a condition analogous to that I have above indicated, and which proved that primitively, in this case, there had been disease of a dental follicle. This was the point of departure for the secondary lesion, which progressively extended all along the side of the jaw, characterized, like inflammation of the bone, by ampliation of the interstices of alveolar osseous tissue, and the hypertrophic development of the organic substance in it. This last, by its predominance over the calcareous element, in which it constituted a kind of myeloplastic substance, explains the extreme fragility of the bone which could be broken by the slightest pressure.

The lymphatic ganglions, raised with the tumor to which they were attached, examined under the microscope by M. Broca, presented the characteristics of a simple adenite.

I do not know of any case that presents any points of analogy to the one just described. It justifies the double point of view, in which we have considered the lesion of the bone by which it was effected. Having in two distinct terms, the first of which corresponds to the original inclusion of the tooth, and the second, to the disease of the bone, this lesion naturally presents two pathological states, of which the last was the most serious, requiring a mutilation of the face which might have been avoided by a recognition of the dental cyst.

Plate 5.—Fig. 1.

Fig. 2.

PLATE 6.

RESUME.—*Diagnosis.—Treatment.*

The diagnosis of the diseases of the maxillary bone, which have been for the most part comprised under the vague name of *osteosarcoma*, has long been undecided, confused, and subject to numerous errors.

A profound study of the histological elements of these diseases has enabled pathological anatomy of late to give a more exact idea of their nature. This, taken in connection with clinical teachings, has determined the difference between them, and subordinated their treatment to the anatomical varieties which mark them. It has also been the design of the present work, and one I hope I have attained, to throw some light on a question of pathology, hitherto obscure, and to open the way to a more severe therapeutic induction.

Thus, in resuming at the symptomatic point of view, the cases contained in this memoir, we find one constant and primordial fact—the absence of one or more of the teeth, which have at no time taken a place in the dental arch. This leading circumstance, ordinarily accompanied by very obstinate neuralgia is due to the displacement and to the elongation of the dental nerves deviating from their proper direction. This results more or less immediately in the formation of an intramaxillary cyst which may sooner or later appear exteriorly under the form of an osseous tumor. Then, when this is manifest with the other antecedents, the surgeon is the more authorized to act promptly, as, in attacking the evil at its root, his intervention should have in view to prevent the ulterior consequences, which, although variable in extent and in gravity, have all, nevertheless, the common character of causing a development of the bone. Hyperostosis thus produced may be unilateral, or bounded by one of the faces of the bone; juxta-alveolar, or circumscribed by only one of the alveoli; or, circumferential, when it occupies the body of the bone.

The inflammation of the bone, as we have seen the direct cause of it, is condensing or rarifying.

In the first case, it is characterized by the peripheric accumulation of osseous elements in excess; this is particularly evident at the angle of the lower jaw and at the base of its coronoid apophysis in the subject of our ninth case. In the second case, it gives rise to vascularity of the osseous tissue from which its sensibility greatly increases. The excess of nutritive movement following is marked by disaggregation of the calcareous elements and the hypertrophy of the organic substance, which, in some cases forms a truly interlamellar myeloplasma, a result causing the jaw to lose its consistence and become very fragile.

These two pathological conditions terminate by suppuration and give place to the formation of multiplied encysted abscesses; these we have found to co-exist especially with anomalies in the position of the teeth.

The third morbid form, which is more particularly met with in anomalies of nutrition, that is to say, where the dental ostoid constitutes a voluminous tumor, is to be understood by a kind of partial or general deduction of the two tables of the bone which, by the slow reabsorption of the organic elements, terminates by being transformed into a cavity whose walls are often constituted by very thin, compact tissue. It often happens that under the confines of the encysted products all the characters of condensing inflammation of the bone present themselves.

It is to be remarked that, in all of these cases, the disease is developed slowly; for some time stationary, it remains nicely circumscribed to the jaw, and at no period of its evolution does it exercise that deleterious influence on the constitution which appertains to accidental disorders; finally, the most minute anatomical examination has in no case revealed the presence of heteromorphous elements like those common to cancerous affections.

This last circumstance, if more favorable for the prognosis, is not the less interesting in view of the treatment; for if a cancerous production exists, the intervention of the surgeon cannot be too radical, but on the contrary, more moderation and reserve must be practiced in the case of a benign and essentially local lesion where the operation can be confined to the precise limits which circumscribe it. Thus in a disease exclusively bounded by osseous tissue, and the base of the jaw, if not perfectly intact, at least but slightly affected by it, the anatomical researches I have made demonstrate the possibility of preserving the continuity of the bone in most cases, making it a duty on the part of the surgeon not to have recourse at first to the partial amputation of the jaw. It is wrong, however, to conclude from these general remarks that, in order to remedy the divers pathological conditions mentioned in this memoir, we can proceed according to some fixed rule. To formularise absolute precepts necessarily obligatory, when experience presents us with facts so different in form, aspect and gravity, although of a common origin, would be to ignore the particular indications which are found in these individual differences, and which are prescribed by the same nature as those secondary disorders which may be produced, not only in the maxillary bone, the primitive seat of the evil, but also in the surrounding soft parts. Thus in the third case of this memoir, it was only necessary, in order to effect a cure, to remove some foreign bodies; some others, on the contrary, would necessitate a much more grave operation. In this, moreover, the surgeon should remember the important indication due to the excellent researches of M. Flourens, on the regeneration of the osseous tissue, by preserving the periosteum as much as possible in the wound by a kind of previous decortication of the jaw about to be raised.

Finally, the treatment proper for like tumors is not clearly indicated; however attentive in clinical examinations, in all cases the incertitude as to the diagnosis is not removed; the origin and seat of these morbid phenomena in the substance of the osseous tissue, on all sides enveloped, remains very obscure. Now the doubt in such cases is in favor of that prudent and conservative surgery to which the man of art should always render an account.

### *Explanation of the Plates.*

### PLATE I.

Fig. 1.—This figure represents part of the left side of body of the lower jaw; the cavity contains the tumor.

*d.* View of the side of the alveolar border.

*a.* Orifice of the dental canal.

*b.* Showing the bicuspid in the line exposed by the anterior resection.

*c.* Crown of the first molar tooth in regular position.

Fig. 2.—*c.* Crown of a large molar seen by a cut made in the wall of the cyst.

*d.* Bicuspid.

*e.* Bicuspid.

*a.* and *b.* Points of the same wall perforated by the prolongations of the tumor.

*f.* Most elevated point of the tumor,

### PLATE II.

Fig. 1.—*a.* Interior appearance of the tumor.

*b.* Large molar reversed.

*c.* and *d.* Cellulo-fibrous membrane interposed between the cyst and the tumor.

Fig. 2.—Examination of the tumor under the microscope by M. Ch. Robin, (400 diameters.) This portion of the tumor is chiefly composed of dentine, the tubes of which radiate more or less regularly from minute depressions in its substance. These tubes closely approximated to each other in one part of their course, diminish in number, and become more and more minute and ramified as they approach the surface of the tumor.

Fig. 2. *d*, *f*.—They terminate in a fine point towards the line of junction between the ivory and the enamel (*a*, *b*, *c*.) or with the cement (*f*, *g*, *h*.) The presence of the ivory forming the greater part of the tumor, demonstrates its character. *Enamel.*—Another important peculiarity is the presence of the enamel at the surface of the tumor and dipping into the depressions which separate the superficial lobules. This enamel varies in thickness. The section of the tumor represented is taken from that part where the enamel is wedged into the dentine. From microscopic dimension it varies to about one millimetre (one-twentieth of an inch.) It is easily recognized by its straight prisms placed close together, and measuring from $\frac{8}{25000}$ to $\frac{6}{25000}$ of an inch in diameter; at *c*, they are, by the accident of preparation, cut obliquely ; at *b* more transversely, and at that part their characteristic form begins to appear.

*Cement.*—At the bottom of the crevices, and here and there in the thickness of the tumor, near its surface, especially in that portion of it which was surrounded by the maxillary bone, the microscope shows laminæ of different thickness, composed entirely of cement.—Fig. 2, *g*. This is immediately in contact (fig. 2, *f*,) with the ivory, between the masses of which it is interposed ; at some places it nearly and at others quite, reaches the enamel. Fig.

PLATE 3.—FIG. I.

FIG. 2.

FIG. 3.

FIG. 4.

2, *f*, *g*, *h*. Besides these structures, sections of the tumor showed small spaces which were either empty or filled with a grayish or brownish matter. These little orifices from $\frac{2}{250}$ to $\frac{6}{250}$ of an inch in diameter, are separated from each other by about $\frac{2}{25}$ of an inch. The microscope shows that these orifices are continuous, with straight, irregular cavities, sometimes forming long canals, hollowed out in the ivory through which they pass. It also shows that these tubes diverge from the cavity just as the tubes of ivory in a healthy tooth diverge from the pulp cavity. The straight, irregular, more or less elongated canals traversing the tumor, and some of which nearly reach the surface, are nothing else but the pulp cavities of the morbid product, either empty, by dessication, or yet containing the dried up pulp in the form of powder.

### PLATE III.

Fig. 1.—*b*. Osseous tumor attached to a molar tooth. (*a*.)

Fig. 2.—*a*, *b*. Sections of the tooth and the tumor.

Fig. 3.—*a*, *b*. Tumor formed by the abnormal development of the bicuspids.
*a*. Top of the tumor. *b*. Its root.

Fig. 4.—*a*. Cyst in the right branch of the lower jaw.
*b*. A molar tooth enclosed in the osseous tissue.
*c*. External wall of the tumor turned over.

### PLATE IV.

Fig. 1.—Represents a cyst occupying the entire extent of the right side of the lower jaw and its coronoid apophysis. The external wall has been removed, leaving a view of its internal wall.

*a*. The wisdom tooth developed abnormally.

*c*. Dental canal open.

Fig. 2.—Same cyst as that in Plate 6.
*c*, *c*. Three incisor teeth, the canine and the bicuspids deviating from their position and leaning against each other.

### PLATE V.

Right side of the lower jaw affected by *ostéite raréfiante*.

*a*. Large molar tooth encysted in the bone.

*b*. Interior of the cyst.

### PLATE VI.

Fig. 1.—*a*. Incisor tooth reversed and directed towards the intermaxillary symphysis.

Fig. 2.—*c*. Canine tooth developed in the substance of the floor of the nasal fossæ.
*b*. *b*. Section of the maxillary sinus.
*d*. Nasal spine.
*a*. *a*. Posterior border of the nasal fossæ.

### PLATE VII.

Figs. 1 and 2.—Two more examples of anomalies in the position of the teeth.

## Figures and Tables

**Plate 1.—Fig. 1. f1:**
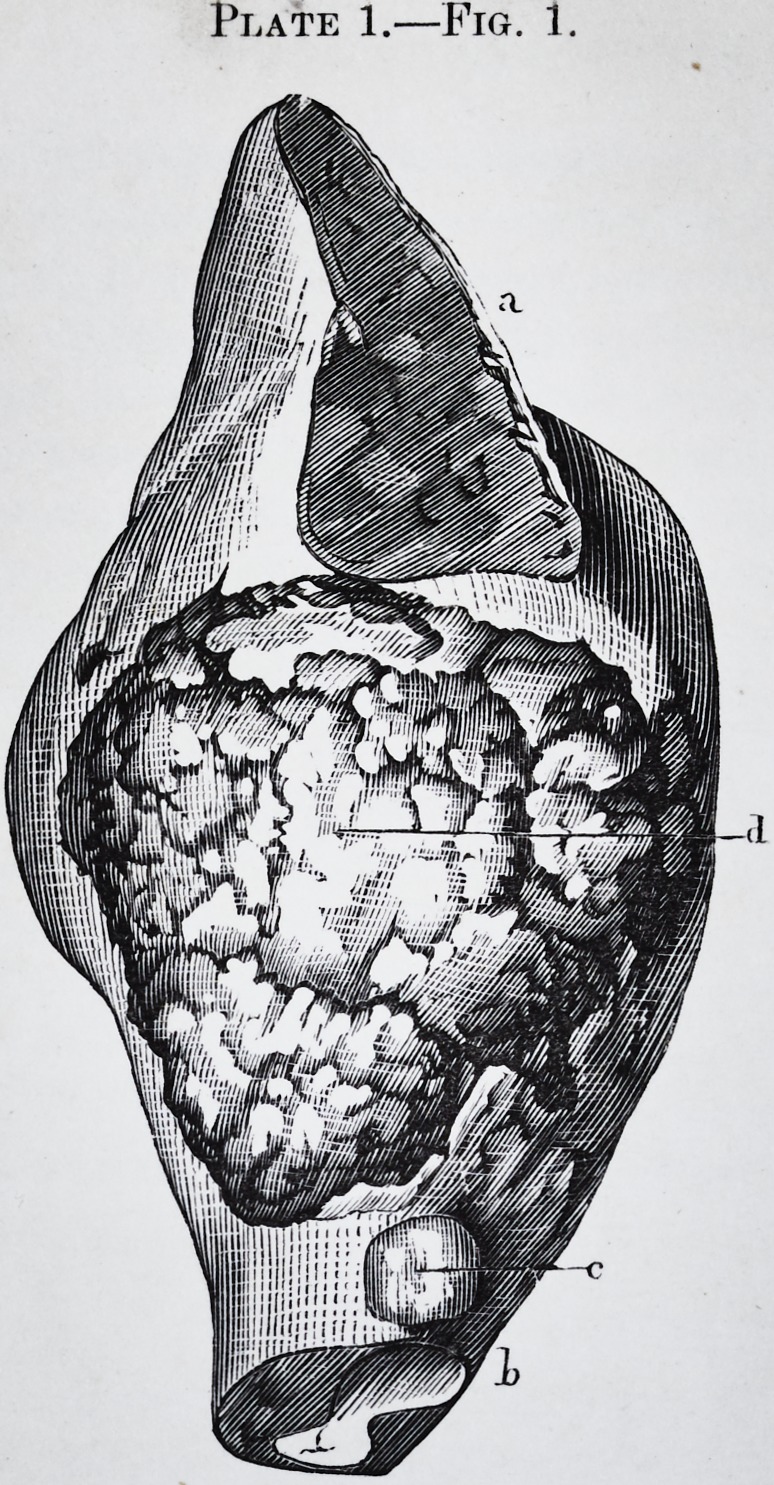


**Fig. 2. f2:**
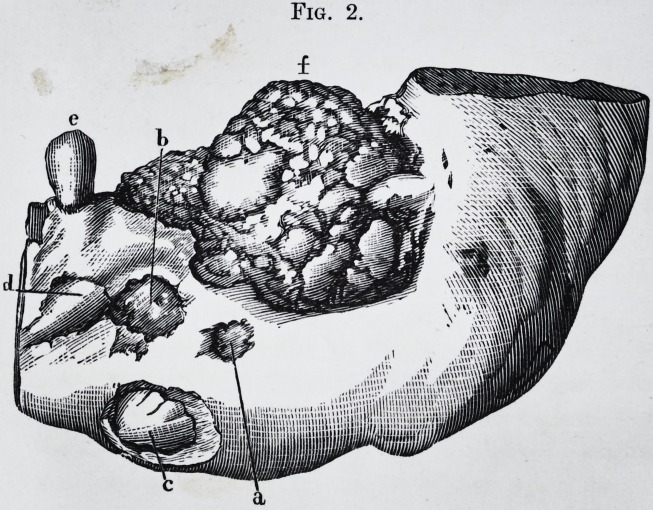


**Plate 2.—Fig. 1. f3:**
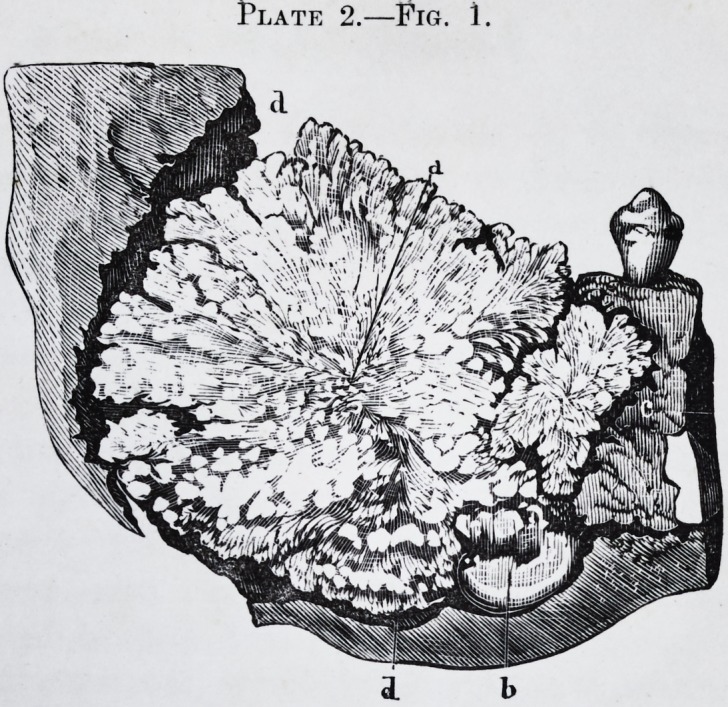


**Fig. 2. f4:**
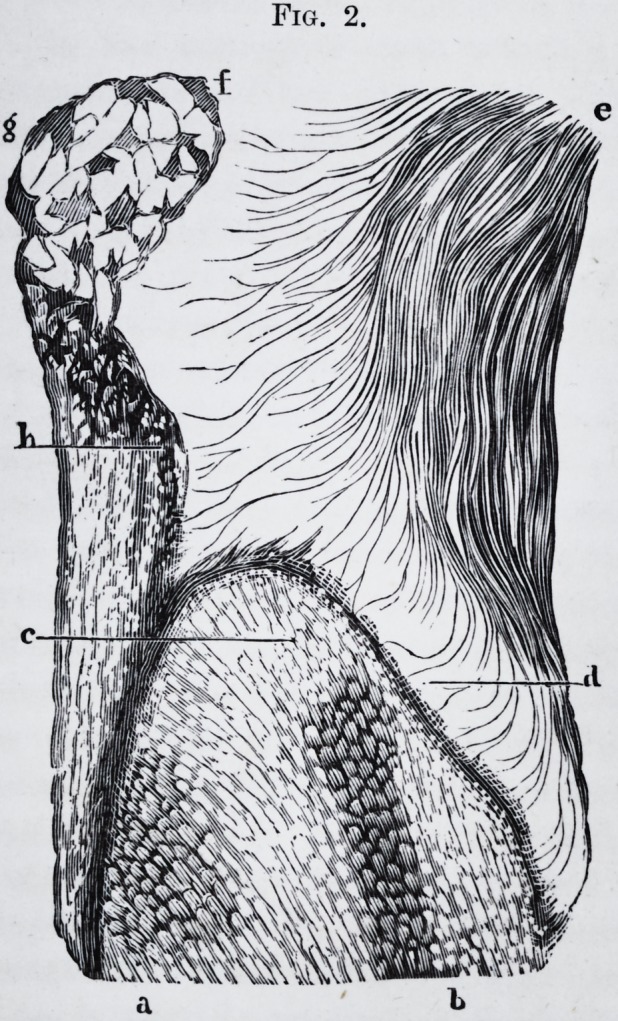


**Plate 7.—Fig. 1. f5:**
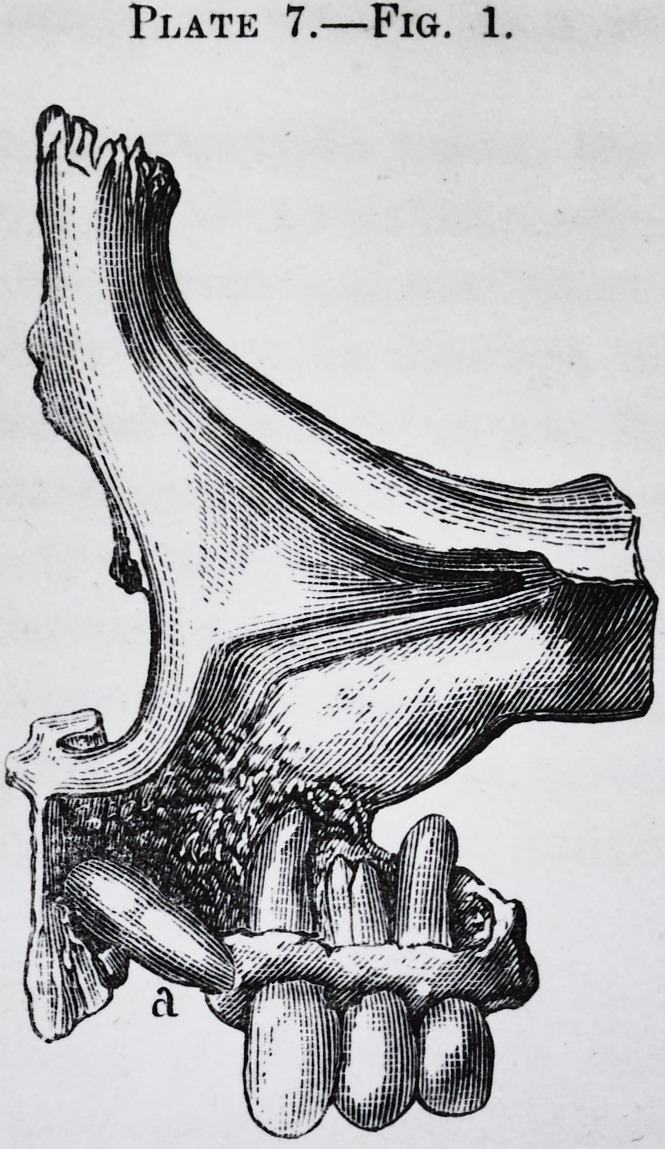


**Fig. 2. f6:**
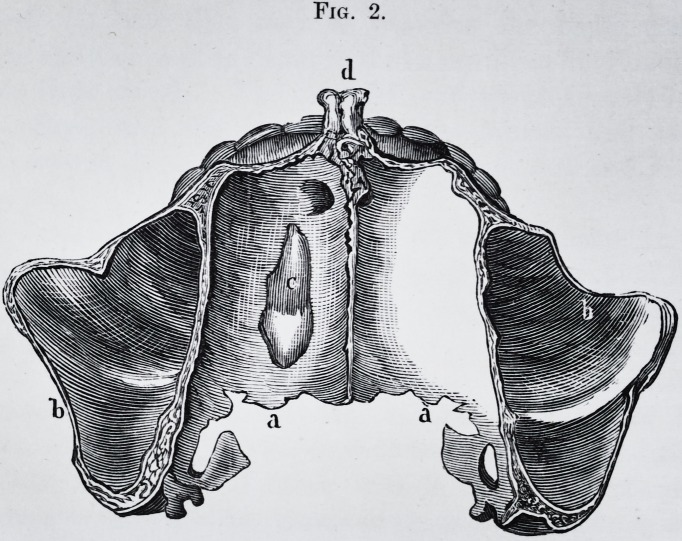


**Plate 8.—Fig. 1. f7:**
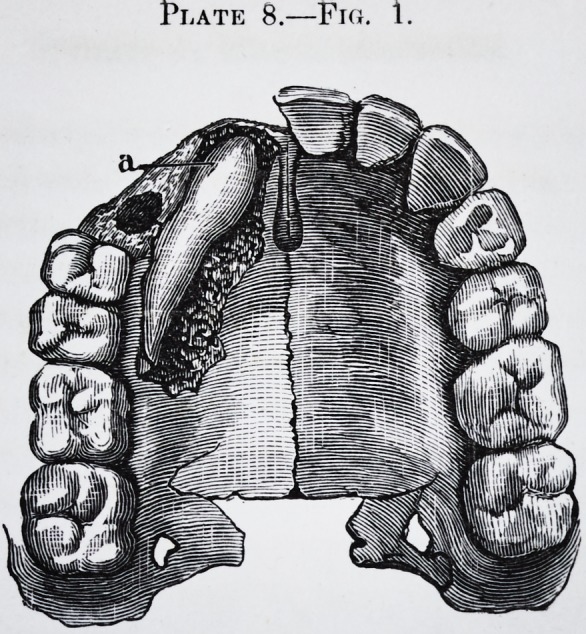


**Fig. 2. f8:**
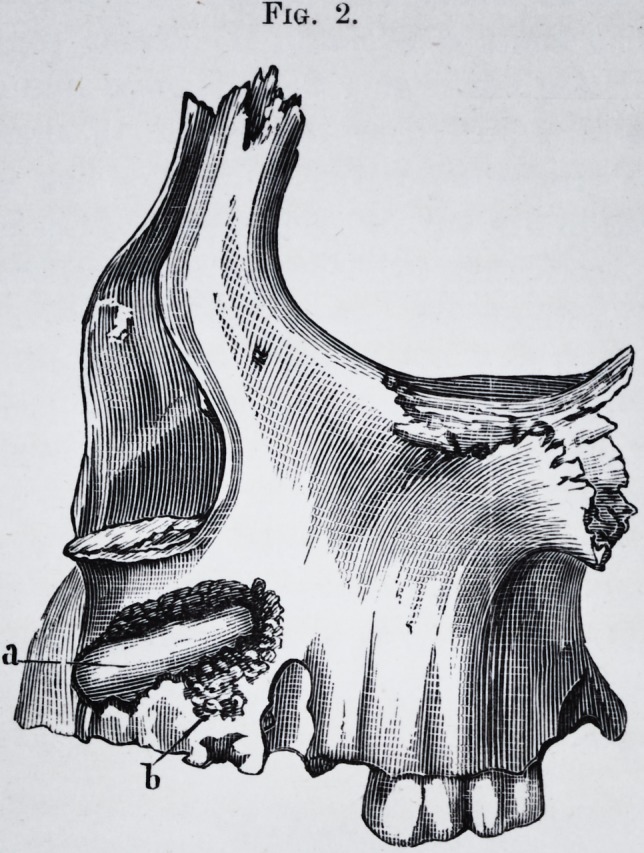


**Plate 5.—Fig. 1. f9:**
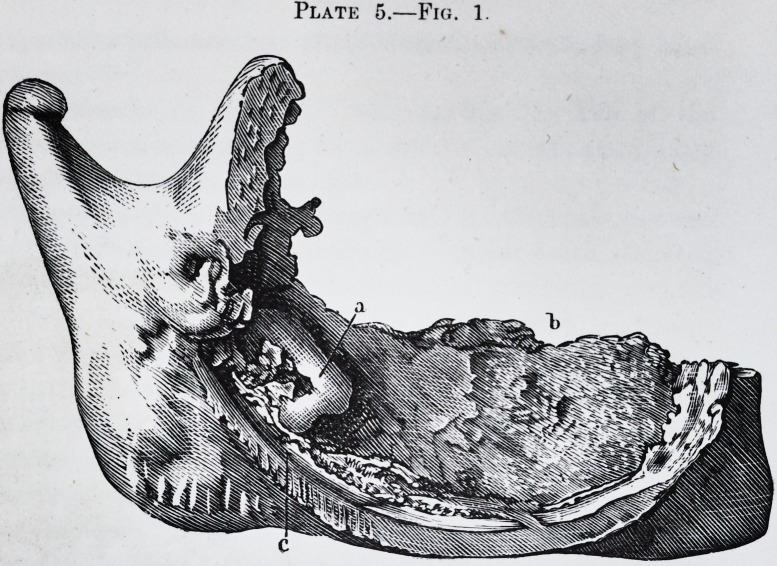


**Fig. 2. f10:**
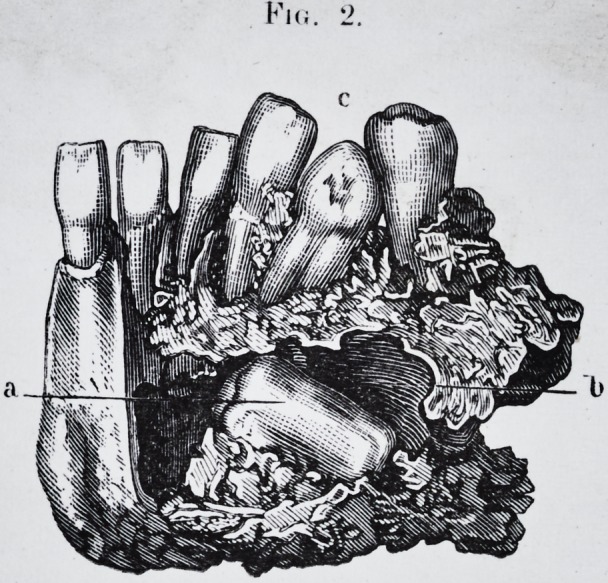


**Figure f11:**
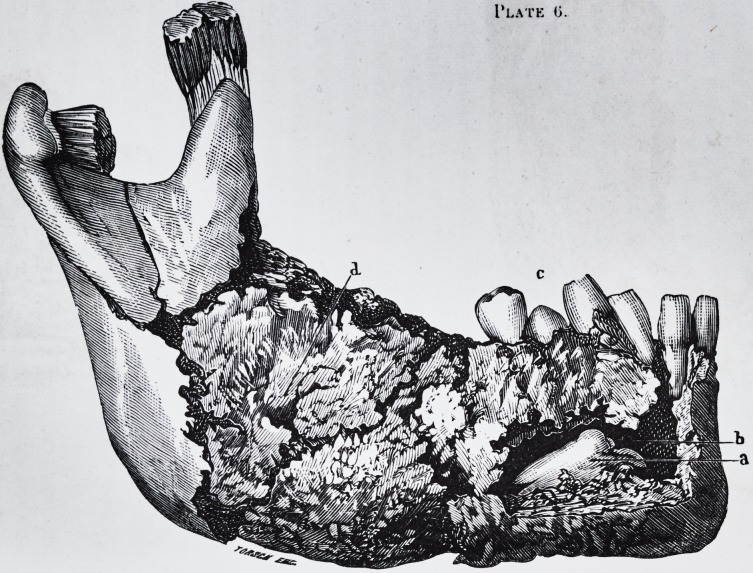


**Plate 3.—Fig. 1. f12:**
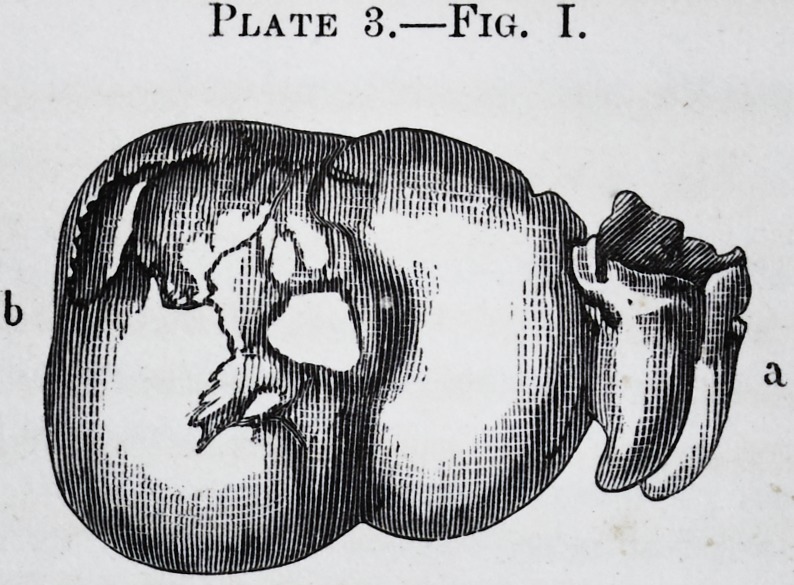


**Fig. 2. f13:**
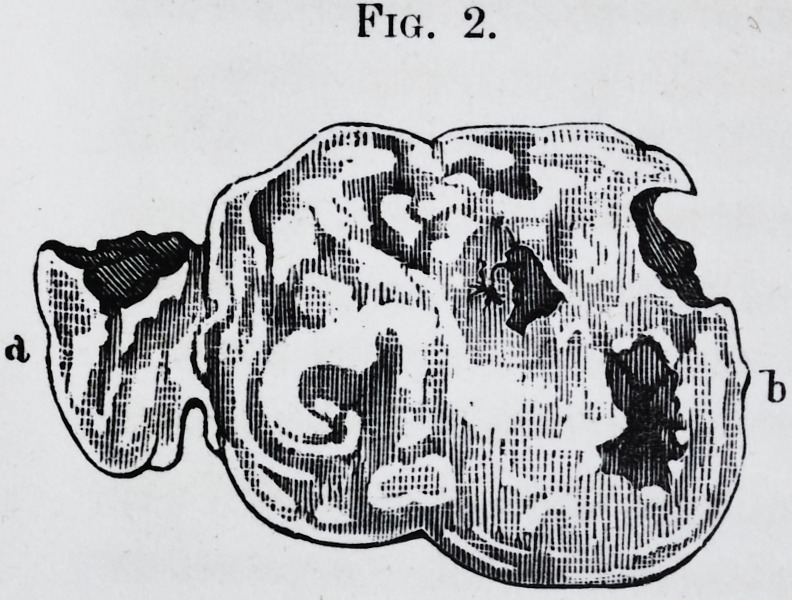


**Fig. 3. f14:**
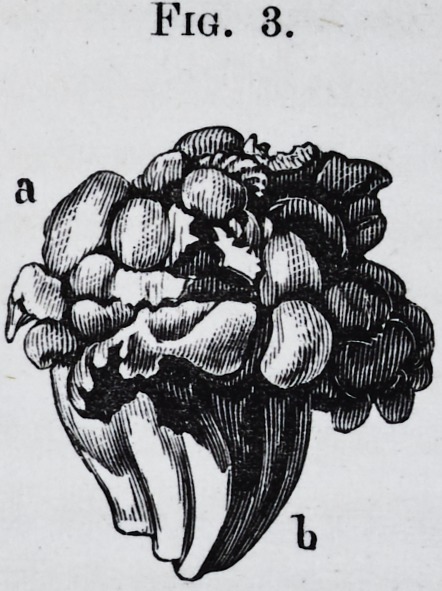


**Fig. 4. f15:**